# Editorial: Application of advances in molecular and genetic researches to tackle pancreatic cancer: screening, diagnosis, prognostication and drug response prediction

**DOI:** 10.3389/fgene.2023.1320082

**Published:** 2023-11-23

**Authors:** Salih Ibrahem, Saleh AlGhamdi

**Affiliations:** ^1^ Department of Basic Medical Sciences, College of Dentistry, University of Kirkuk, Kirkuk, Iraq; ^2^ Research Center, King Fahad Medical City, Riyadh, Saudi Arabia

**Keywords:** pancreatic cancer, molecular, diagnosis, prognosis, drug response

Pancreatic cancer has been called the king of cancer owing to its aggressive and lethal behavior. According to the International Agency for Research on Cancer (IARC) 2020 statistics, pancreatic cancer continues to contribute significantly to the worldwide burden of malignant diseases, ranking as the 12th most prevalent cancer (2.6% of all cancers) and the seventh most common cause of cancer mortality (4.7% of all cancers).

The incidence and mortality rates of pancreatic cancer are widely different; nations with high or very high Human Development Indexes have roughly five times higher rates when compared to low or medium countries. Age affects the incidence rates for both genders, with elderly adults over 70 having the highest rates and people older than 55 accounting for 90% of all incidences of pancreatic cancer.

Moreover, it has a very poor prognosis and its 5-year survival does not exceed 5% of the diagnosed cases in the best scenario. This nightmare prognosis is attributed to various factors such as the aggressive nature, chemoresistance, and late-stage diagnosis. The delay in the diagnosis of pancreatic cancer is attributed to having no specific symptoms, poor public awareness, lack of early screening tests, and difficult pancreatic anatomy which makes even advanced radiological diagnosis difficult.

The molecular signature of pancreatic cancer is mainly centered around a handful bunch of rug genes such as *KRAS*, *P53*, *CDKN24*, *SMAD4*, and *BRCA 1&2*. Unlike many of the other tumors, no druggable-offending gene was found in pancreatic cancer which made molecular treatment not to be on the table.

The above facts have made pancreatic cancer a fertile land for basic, preclinical, and clinical research which gave us the motivation and the courage to allocate a Research Topic in Frontiers in Genetics, aiming to bring together the latest studies in the field.

The first paper by Döring et al. investigated the prevalence of germline pathogenic variants in calcitonin-producing pancreatic neuroendocrine neoplasms in a cohort of five Taiwanese patients using whole genome sequencing.

The study found single nucleotide substitutions and small indels in *ATM*, *BRCA1*, *BRCA2*, *POLQ*, *SPINK1*, and *CASP8* as well as structural variants in *CDC25C* and *USP44*. This combination of variants makes a special molecular signature for this specific tumor that might be used in the future for prognostic and therapeutic purposes. The authors argue that germline mutations have a larger contribution to the pathogenesis of the disease. Despite the importance of this work, its scientific value might have been affected by its small sample size.

Immunotherapy has been a hotspot for cancer research. Research conducted by Zhu et al. investigated the role of Krüppel-like factor 3 (KLF3) which is a key transcriptional repressor in the microenvironment of pancreatic cancer. This study is, in part, database-dependent, in addition to *in vivo* (mouse model) and *in vitro* (cell line) work. The outcome of the study showed that KLF3 was involved in various immune and progression-related pathways since its expression is higher in tumors than in normal tissues and even higher in aggressive tumors. Its silencing was associated with a slower rate of tumor progression. Indeed, its high expression is associated with poor response to immunotherapy. This marker opens the door to immunotherapy in pancreatic cancer and might be used as part of a prognostic system.

Guo et al. tried to establish prognostic and predictive scores for pancreatic ductal carcinoma that utilized Ubiquitination-related genes (URGs). URGs have been reported as regulators of tumors, affecting tumor cell cycle regulation, gene expression, and progression.

The authors used two cancer databases, the Cancer Genome Atlas (TCGA) and the International Cancer Genome Consortium (ICGC), to establish prognostic and predictive systems for pancreatic ductal carcinoma in addition to cell culture work for functional studies. A special system was used to establish URGs’ prognostic signature depending on three Ubiquitination-related genes (*SLC22A17*, *UCHL1*, and *S100A2*) that are involved in many cancer pathogenesis.

The research group summarized their research conclusion (we designed a molecular cluster and prognostic signature based on URGs, which aid in anticipating survival, directing immunotherapy, and determining clinical outcomes. This research potentially provides deeper insights into the function of ubiquitination in PDAC and facilitates the development of more effective therapies for this disease).

Chung et al. think that germline mutations have a greater contribution to PDAC and previous studies have underestimated their role due to the selection of coding areas of a limited number of genes. In the current work, the authors selected 24 Taiwanese patients and performed genome sequencing for their peripheral blood cells for 750 genes with diverse genetic variant types such as SNP, small insertions and deletions and inversions. Paired-end reads were aligned to the reference genome (GRCh38/hg38) using BWA-MEM to identify germline mutations.

Almost one-third of the cases had germline mutation in mainly five genes *ATM*, *BRCA1*, *BRCA2*, *POLQ*, and *SPINK1* with an unequivocal role in pancreatic cancer pathogenesis. The remaining of the 750 studied genes were not of strong contribution to PDAC. The study emphasized the role of these mutations in the pathogenesis of cancer and might be a future prognostic and predictive molecular candidate.

